# Editorial: Epitranscriptomics: The Novel RNA Frontier

**DOI:** 10.3389/fbioe.2018.00191

**Published:** 2018-12-04

**Authors:** Giovanni Nigita, Mario Acunzo, William Chi Shing Cho, Carlo M. Croce

**Affiliations:** ^1^Department of Cancer Biology and Genetics, The Ohio State University, Columbus, OH, United States; ^2^Division of Pulmonary Diseases and Critical Care Medicine, Virginia Commonwealth University, Richmond, VA, United States; ^3^Department of Clinical Oncology, Queen Elizabeth Hospital, Kowloon, Hong Kong

**Keywords:** epitranscriptomics, RNA methylation, RNA editing, psuedourylation, RNA

From the formulation of the Central Dogma of molecular biology to the discovery of novel non-coding RNA (ncRNAs) classes, the focus of Genetics involved DNA variants with the scope of elucidating biological pathways perturbed in disease. Recently, considerable attention has shifted toward the study of RNA modifications dynamically occurring in both protein-coding as well as non-coding RNAs, in both animal and plant kingdoms. This, in turn, has gradually led to the exciting exploration of the novel frontier of “Epitranscriptomics,” revealing an additional, finer layer of complexity in gene regulation.

The application of the fast-growing high-throughput sequencing technology (HTS) has allowed the unprecedented opportunity to unveil millions of RNA modifications in human genes, such as resulting from methylation (e.g., m6A, m1A, m5C, hm5C, 2'OMe), pseudourylation (Ψ) and deamination (e.g., A-to-I RNA editing), counting to date more than 140 different forms of RNA modifications.

The aim of this Research Topic was to collect both original research articles and reviews in addressing the bioinformatics approaches and wet lab methodologies necessary to aid in the detection of novel RNA modifications and characterization of their biological functions, as well as contribute to the identification of the molecular protagonists involved in the regulation of such phenomena. Among the articles embracing the scope of the Research Topic, we have collected five reviews, four original research and methods articles, and a technology article.

Two reviews focused on RNA modifications in ncRNAs. Romano et al. surveyed the main types of RNA methylation in the context of ncRNAs with their associated functions. They also described the methodologies to identify and profile such RNA modifications. On the other hand, Zhao et al. instead, focused on the role of the pseudouridylation of ncRNAs in nuclear gene expression events. In particular, they reviewed how, in the nuclear context, ncRNA pseudouridylation contributes to transcription and pre-miRNA splicing.

Another important RNA modification is the non-templated addition of uridine(s) to the terminal end of RNA, termed 3′ RNA uridylation, which has been described having a role in the regulation of both mRNAs and ncRNAs. In this collection, Menezes et al. reviewed current discoveries of the functional roles for 3′ RNA uridylation, particularly focusing on mammalian biology.

RNA modifications add a novel and fine-tuning layer to gene expression regulation, having recently shown how the dysregulation of these phenomena can lead to the onset of disease, such as neurological diseases and cancer. In this collection, Angelova et al. discussed the emerging findings that show indicate the modifications of RNA impact the development of the brain and their involvement in neurological disorders. One of the most abundant internal modifications in messenger RNAs (mRNAs) is represented by N6-methyladenosine (m6A), which has been discovered to play important roles in multiple biological processes. Liu et al. reviewed how the malfunctions of m6A machineries lead to several cancer types, including solid and non-solid tumors.

RNA modification phenomena are also present in the plant kingdom. It has been observed that post-transcriptional modifications, such as C-to-U RNA editing events, can occur in chloroplasts. In this collection, Rodrigues et al. introduced a method to profile RNA editing from chloroplast by using small RNA (sRNA) libraries form *Arabidopsis*, soybean and rice. The results obtained in this study encourage using sRNA libraries in order to identify novel RNA editing events and confirm previous detected ones, as well as profiling RNA editing in different plants under different biological conditions.

The improvements of high-throughput sequencing (HTS) techniques allowed us to unveil thousands of editing events in plants. In this collection, Lo Giudice et al. presented the third release of REDIdb, a freely available database for RNA editing events in plants organelles. The current release of REDIdb contains more than 26K RNA editing modifications, together with a new web interface allowing users to contextualize editing events in their genomic, biological and evolutionary contexts.

Together with the improvements of HTS technologies, we have observed remarkable progress in the development of algorithms and computational methods able to analyze and study data in the emerging field of Epitranscriptomics. Song et al. presented PEA-m5C, a transcriptome-wide machine learning-based predictor of m^5^C modification in *Arabidopsis*. PEA-m5C was developed by employing a random forest algorithm with sequence-based features and optimized window, obtaining an average AUC of 0.939 in 10-fold cross-validation experiments.

The collection also includes a study conducted in Soybean seedlings by Chmielowska-Bak et al. concerning the influence of short-term cadmium stress on two RNA oxidation-dependent modifications: 8-hydroxyguanosine and apurinic/apyrimidinic sites. The authors reported an increased level of RNA oxidation in plants in stress conditions.

Finally, the collection also includes a study on the effect of centrifugal force in quantification of colorectal cancer-related mRNA in plasma using targeted sequencing. We know that the pre-analytical factors are critical for the measurement of analytes in the blood. This study investigated two common centrifugation protocols on plasma mRNA quality and quantity. The authors concluded that more targeted mRNAs could be found by double centrifuges of 1,600xg followed by 16,000xg than a single centrifuge of 3,500xg.

Epitranscriptomics represents a thrilling, vast and novel field in Cellular Biology. The articles we finally accepted for our Research Topic address some of its most exciting challenges, with the intention of providing a useful resource that will peak the interest of many researchers investigating the RNA modification phenomena. We would like to take this opportunity to thanks the contributions of the authors and the professional support from the editorial staffs.

## Author Contributions

All authors listed have made a substantial, direct and intellectual contribution to the work, and approved it for publication.

### Conflict of Interest Statement

The authors declare that the research was conducted in the absence of any commercial or financial relationships that could be construed as a potential conflict of interest.

